# A NEW APPROACH FOR HEMORRHOID DISEASE: SELECTIVE DEARTERIALIZATION AND MUCOPEXY WITHOUT DOPPLER GUIDANCE

**DOI:** 10.1590/0102-672020210001e1560

**Published:** 2021-05-14

**Authors:** Carlos Walter SOBRADO, Lucas Faraco SOBRADO, Sergio Carlos NAHAS, Ivan CECCONELLO

**Affiliations:** 1 Digestive and Colorectal Surgery Division, Department of Gastroenterology, Hospital das Clínicas, University of São Paulo School of Medicine, São Paulo, Brazil

**Keywords:** Transanal minimally invasive surgery, Transanal hemorrhoidal dearterialization, Hemorrhoids, Mucopexy, Surgical technique, Desarterialização hemorroidária transanal, Hemorroidas, Mucopexia, Complicações, Técnica cirúrgica

## Abstract

**Background:**

Transanal hemorrhoidal dearterialization (THD) is safe and effective minimally invasive treatment for hemorrhoidal disease, but reports regarding recurrence and postoperative complications (pain and tenesmus) vary significantly.

**Aim:**

To evaluate if selective dearterialization and mucopexy at the symptomatic hemorrhoid only, without Doppler guidance, achieves adequate control of the prolapse and bleeding and if postoperative morbidity is reduced with this technique.

**Methods:**

Twenty consecutive patients with grade II and III hemorrhoids were treated with this new approach and were evaluated for postoperative complications and recurrence.

**Results:**

Control of prolapse and bleeding was achieved in all patients (n=20). Postoperative complications were tenesmus (n=2), external hemorrhoidal thrombosis (n=2) and urinary retention (n=2). After a mean follow-up of 13 months no recurrences were diagnosed.

**Conclusion:**

Selective dearterialization and mucopexy is safe and achieves adequate control of prolapse and bleeding and, by minimizing sutures in the anal canal, postoperative morbidity is diminished. Doppler probe is unnecessary for this procedure, which makes it also more interesting from an economic perspective.

## Graphical abstract

[Fig f1a]

**The surgical f1a:**
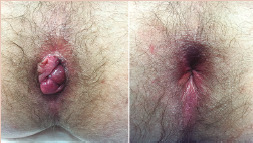
procedure: three dearterializations and three mucopexies

[Fig f1as]

**O procedimento f1as:** cirúrgico: três desarterializações e três mucopexias

## RESUMO

**Racional:**

O tratamento da doença hemorroidária pela técnica de THD (Transanal Hemorrhoidal Dearterialization) é minimamente invasivo e tem se mostrado seguro e eficiente. No entanto, dados sobre a recorrência e complicações (dor e tenesmo) no pós-operatório são muito variáveis.

**Objetivo:**

Avaliar se a desarterialização e mucopexia seletiva, sem o uso de Doppler, é suficiente para o controle de sintomas e se a morbidade pós-operatória é menor com esta técnica.

**Métodos:**

Vinte pacientes foram tratados com essa técnica e avaliados sobre controle de sintomas, morbidade pós-operatória e recorrência.

**Resultados:**

Controle do prolapso e sangramento foi observado em todos pacientes (n=20). Complicações pós-operatórias foram: tenesmo (n=2), trombose hemorroidária externa (n=2), retenção urinária (n=2). Após um seguimento médio de 13 meses, nenhuma recorrência foi detectada.

**Conclusões:**

O procedimento de desarterialização e mucopexias seletivas é seguro e eficiente em termos de controle do prolapso e sangramento. Esta técnica resulta em menor morbidade cirúrgica, uma vez que diminui o número de suturas no canal anal, resultando em menos dor e tenesmo pós-operatório. Para este procedimento o uso de ultrassom Doppler é desnecessário, o que diminui custos e o torna mais atrativo do ponto de vista econômico.

## INTRODUCTION

Hemorrhoidal disease is highly prevalent and several treatment options are currently available. Pile excision has generally been regarded as the gold standard treatment but is associated with intense postoperative pain^10^. Minimally invasive techniques have thus gained wide acceptance among colorectal surgeons and their patients due to the lower morbidity.

In 1995, Morinaga et al.^3^ described the hemorrhoidal artery ligation (HAL) procedure, in which the distal branches of the superior rectal artery were ligated - later renamed as transanal hemorrhoidal dearterialization (THD). More recently, different authors have proposed technical adjustments such as the addition of mucopexy^2^ and the concept of distal Doppler dearterialization^4^, aiming to achieve a more precise ligation of the branches of the superior rectal artery.

To date, the surgical technique has not been standardized and different surgeons perform different number of dearterializations and mucopexies - data that are often omitted from clinical trials evaluating this technique. This may explain the heterogeneity of outcomes in terms of postoperative morbidity, and possibly also in recurrence.

In this paper we propose technical adjustments in the THD-mucopexy technique that have improved our results. Our impression is that minimizing the number of sutures in the anal canal, making dearterializations and mucopexies only symptomatic haemorrhoids, achieves similar good outcomes as the original technique, but less postoperative morbidity, especially pain and tenesmus. This may be the next step in this minimally invasive procedure.

## METHODS

Between July 2018 and December 2019, 20 patients were recruited for THD. Selective dearterialization and mucopexy were performed without Doppler guidance. All surgeries were performed by the same surgeon (CWS).

### Operative technique

**FIGURE 1:**
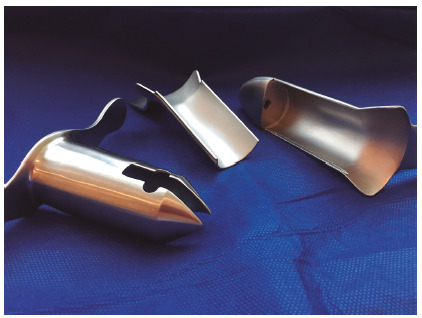
Reusable pivot proctoscope

For the THD-mucopexy procedure, the patient is placed in the lithotomy position either with spinal or general anesthesia. Intravenous ciprofloxacin is administered as a single dose. Following lidocaine gel lubrification, anuscopy is performed with a pivot proctoscope to evaluate for internal hemorrhoids. Our preference is to perform this procedure with a reusable proctoscope in order to reduce costs (Figure 1).

**FIGURE 2:**
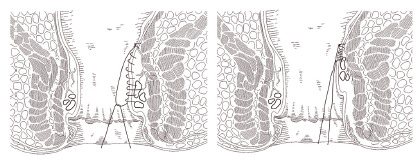
Schematic drawing of the dearterialization and mucopexy suture

**FIGURE 3:**
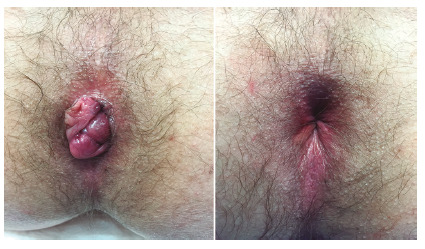
Intraoperative picture showing before and after the surgical procedure (three dearterializations and three mucopexies)

Following the identification of the hemorrhoids, a longitudinal continuous suture (mucopexy) starting 4-5 cm above the anal verge is performed, until just above the visible pathologic pile, using a 2-0 absorbable polyglycolic acid with a 5/8-inch needle. The depth of the suture is calibrated as to include the mucosa and submucosa of the rectal wall, which is better achieved with the guidance of a pivot proctoscope. The passages of the needle should not be more than 5 mm apart in order to avoid long gaps in the suture line and to avoid excessive tension. The knot is tied so that the prolapsed pile is lifted in the direction of the distal rectum as schematic depicted in Figure 2. The postoperative result is shown in Figure 3.

The inclusion of the hemorrhoidal pile in the suture depends on the degree of the prolapse. If reduction cannot be achieved with standard mucopexy, then the upper portion of the pile should be included in the suture - more often for mixed hemorrhoids. It should be noticed that extension of the suture beyond the pectinate line is not recommended as it yields increased postoperative pain.

## RESULTS

Patient’s demographics are summarized in Table 1.

**TABLE 1:** Patient’s demographics

Age (range)	19 - 69 years old
Gender	Male (n=12) Female (n=8)
Goligher’s classification	Grade II (n=2) Grade III (n=18)
Symptoms	Prolapse (n=20) Bleeding (n=18) Itching (n=10)
Postoperative follow-up (mean)	13 months (7-18)
Operative time (mean)	19-27 min (22 min)
Days to return to work	4 days (n=2) 5 days (n=12) 7 days (n=4) 10 days (n=1) 14 days (n=1)

Control of bleeding and prolapse was achieved in all patients. No recurrences were detected after a mean follow-up of 13 months. The number of dearterializations and mucopexies sutures were four (n=13), three (n=6) and two (n=1). Associated procedures were necessary in three patients, resection of skin tag (n=2) and hyperthrophic papilla (n=1).

Postoperative complications included tenesmus (n=2), urinary retention (n=2) and external hemorrhoidal thrombosis (n=2). No patients required opioids for pain control. Severe complications such as anal stenosis, faecal incontinence or abscess were not reported until the writing of this manuscript. Most of the patients were very satisfied (n=18) with the procedure, one was somewhat satisfied and one was indifferent (both patients who had postoperative external hemorrhoidal thrombosis). Return to work or normal daily activity occurred until the 7^th^ postoperative day for 18 patients (90%).

## DISCUSSION

Hemorrhoidal disease has been historically associated with intense postoperative pain, which has frightened patients for centuries. In 1995, Morinaga et al^3^ described a minimally invasive technique for the treatment of hemorrhoidal disease, in which he ligated the distal branches of the superior rectal artery guided by a Doppler-probe, which was named “hemorroidal artery ligation” (HAL) and achieved control of the prolapse in 72% of patients. In an attempt to reduce recurrence, Dal Monte et al^2^ added longitudinal sutures at the distal rectum (mucopexy) for prolapse piles and achieved control of prolapse in 92% of patients. 

Ligation of the distal branches of the superior rectal artery has traditionally thought to play an important role in the good results achieved with the THD technique. However, studies of the anatomy of the anal canal have shown a great variability in terms of vascular anatomy of hemorrhoids and have questioned if the ligation of a specific artery is the main mechanism by which THD works^1^ ^,^ ^6^.

Ratto et al.^4^ suggested that the dearterialization should be done distally in the rectum, as to include the branches of the superior rectal artery in the position where they were most superficial in relation to the mucosa, and named it distal Doppler dearterialization.

Brazilian multicentre study of 705 patients reported control of bleeding and prolapse in 97.9% and 93.6% of patients respectively, but 21.4% complained of tenesmus and 7.2% had opioid-requiring pain^9^. Another study conducted by our group particularly compared total mucopexy and partial mucopexy (less than six sutures) guided by Doppler and found a much higher rate of postoperative pain, tenesmus and fecal impaction in the former group^9^. It is reasonable to consider that more sutures in the anal canal results in more early postoperative morbidity, possibly due to tissue disruption and local edema.

In some series, the number of dearterializations required to silent all Doppler signals ranged from two to over 10, suggesting a high variability in local vascular anatomy^5^. Randomized controlled trials compared THD with and without Doppler probe and achieved similar results, suggesting that targeting a specific artery and the distal rectum is unnecessary^7^. Cadaveric studies found an average of eight branches of the superior rectal artery in the distal rectum and their locations were not at 3’, 7’ and 11’ o’clock as classically described^6^.

Currently, we perform selective dearterialization and mucopexy at the visible pile only, which is basically adapting the concepts of traditional excisional hemorrhoidectomy to the new minimally invasive THD procedure and only treating the symptomatic pile. 

In this present study, none of the patients required opioid for pain control and we had 10% of postoperative tenesmus, which is a substantial improvement in comparison to our previous results with complete dearterialization and mucopexy guided by Doppler, in which 26% of patients had tenesmus and 14% required opioids for pain control^8^.

Long-term results and large series with this approach still have to be assessed.

## CONCLUSION

This new approach for transanal hemorrhoidal dearterialization constitutes of selective dearterialization and mucopexy, only in symptomatic haemorrhoids with prolapsed. This technical standardization with less sutures in the anal canal, results in less postoperative morbidity, especially pain and tenesmus. Doppler probe is unnecessary for this procedure, which makes it also more interesting from an economic perspective. 
